# Genome Diversity and the Origin of the Arabian Horse

**DOI:** 10.1038/s41598-020-66232-1

**Published:** 2020-06-16

**Authors:** Elissa J. Cosgrove, Raheleh Sadeghi, Florencia Schlamp, Heather M. Holl, Mohammad Moradi-Shahrbabak, Seyed Reza Miraei-Ashtiani, Salma Abdalla, Ben Shykind, Mats Troedsson, Monika Stefaniuk-Szmukier, Anil Prabhu, Stefania Bucca, Monika Bugno-Poniewierska, Barbara Wallner, Joel Malek, Donald C. Miller, Andrew G. Clark, Douglas F. Antczak, Samantha A. Brooks

**Affiliations:** 1 Department of Molecular Biology and Genetics, Cornell University, Ithaca, NY 14853 USA; 2 Baker Institute for Animal Health, College of Veterinary Medicine, Cornell University, Ithaca, NY 14853 USA; 3 Department of Animal Science, UF Genetics Institute, University of Florida, Gainesville, FL 32610 USA; 4 Department of Animal Science, College of Agriculture and Natural Resources, University of Tehran, Karaj, Iran; 5 Department of Cell and Developmental Biology and Biochemistry, Weill Cornell Medical College in Qatar, Doha, Qatar; 6 Present Address: Prevail Therapeutics; New York, New York, 10016 USA; 7 Department of Veterinary Science, Maxwell H. Gluck Equine Research Center, University of Kentucky, Lexington, KY 40546 USA; 8 Department of Animal Reproduction, Anatomy and Genomics, University of Agriculture in Kraków, Kraków, Poland; 9 Equine Hospital at Sha Tin Racecourse, The Hong Kong Jockey Club, Hong Kong, China; 10 Equine Veterinary Medical Center, Member of Qatar Foundation, Doha, Qatar; 11 Institute of Animal Breeding and Genetics, University of Veterinary Medicine Vienna, Vienna, 1210 Austria; 12 Department of Genetic Medicine, Weill Cornell Medical College in Qatar, Doha, Qatar

**Keywords:** Genetics, Physiology

## Abstract

The Arabian horse, one of the world’s oldest breeds of any domesticated animal, is characterized by natural beauty, graceful movement, athletic endurance, and, as a result of its development in the arid Middle East, the ability to thrive in a hot, dry environment. Here we studied 378 Arabian horses from 12 countries using equine single nucleotide polymorphism (SNP) arrays and whole-genome re-sequencing to examine hypotheses about genomic diversity, population structure, and the relationship of the Arabian to other horse breeds. We identified a high degree of genetic variation and complex ancestry in Arabian horses from the Middle East region. Also, contrary to popular belief, we could detect no significant genomic contribution of the Arabian breed to the Thoroughbred racehorse, including Y chromosome ancestry. However, we found strong evidence for recent interbreeding of Thoroughbreds with Arabians used for flat-racing competitions. Genetic signatures suggestive of selective sweeps across the Arabian breed contain candidate genes for combating oxidative damage during exercise, and within the “Straight Egyptian” subgroup, for facial morphology. Overall, our data support an origin of the Arabian horse in the Middle East, no evidence for reduced global genetic diversity across the breed, and unique genetic adaptations for both physiology and conformation.

## Introduction

The Arabian is the oldest recorded breed of horse, with credible documentation and pictorial representations extending back at least 2,000 years that place the development of the breed in the Middle East region^1–3^. Paleogenomic evidence supports the contribution of ancient Persian lineages during the early formation of modern European horse breeds around 1100-1300 YA^4^. The horse of the desert further expanded alongside the rise of the nomadic Bedouin tribes who valued these horses as a cultural symbol, source of wealth, and a military resource^2^. Today, although surpassed in absolute numbers by the American Quarter Horse, the Arabian breed is still the most widespread around the world^5^, with pedigree registries in at least 82 countries (http://www.waho.org).

The modern Arabian horse possesses a unique conformational phenotype that includes a dish-shaped facial profile, wide-set eyes, arched neck, and high tail carriage^6^. However, Arabian horses in photographs made in the late 1800s and early 1900s often show less pronounced facial dishing and lower tail carriage^2^, suggesting that these traits may be under strong selection by modern Arabian breeders, particularly for lines of horses used primarily for non-ridden show competitions. The Arabian horse is also renowned for its heat tolerance and athletic endurance, making the Arabian a popular breed for long-distance races, where they carry the weight of a rider across distances of up to 160 km in winning times of around 8 hours^7^. Analysis of Arabian endurance horses has indicated that predisposition for this type of athletic competition is a multi-genic trait^7^.

Arabian horses have been exported from their ancestral homeland for several centuries. However, the exported populations usually had small numbers of founder animals, and consequently, now may have limited genetic diversity^8^. Considered together with the aforementioned selection for conformational phenotype, the likelihood of deleterious inbreeding in Arabians should be high, and it is not surprising that several important autosomal recessive inherited diseases have been identified in Arabians^9,10^. In contrast, the few studies of diversity of Arabian horses in the Middle East have shown higher levels of variation in these horses compared to the progeny of exported Arabians in other parts of the world^11–13^. Despite the evidence for antiquity of the Arabian breed, there is relatively little solid documentation for the various strains and maternal lineages of Arabian horses that are maintained by Arabian horse fanciers and breeders^2^. In fact, several molecular studies using mitochondrial DNA have failed to confirm the traditional Arabian horse maternal lineages transmitted by oral histories^14–17^.

For over 100 years the influence of Arabian horses in ‘improving’ other horse breeds has been generally accepted among horsemen^18^. The best-documented example of such influence is in the pedigree of the Thoroughbred breed, which has been maintained as a Stud Book since 1791^19^. In a pedigree-based analysis of founder lines of the Thoroughbred, Cunningham and colleagues found that three stallions imported to England from the Middle East around the turn of the 18^th^ century remain major contributors to the modern-day Thoroughbred gene pool: the Godolphin Arabian (sometimes termed a Barb), estimated to contribute 13.53% of modern gene pool by pedigree analysis, as well as the Darley Arabian, and the Byerley Turk^20^. Recently however, an analysis of horse Y chromosome haplotypes has indicated that the Y haplotype of the “Darley Arabian” actually originated from the Turkoman horse, an ancient breed from the Middle East and Central Asia that is like the Arabian Horse, also an “Oriental” type breed^21^. This calls into question the role of the Arabian as a founder of the Thoroughbred breed, and more generally, to its influence on other horse breeds.

This study documents population structure across a large global sampling of the Arabian breed of horse. We used an equine single nucleotide polymorphism (SNP) array and whole-genome re-sequencing in a comprehensive genome-wide analysis of genetic diversity of Arabian horses that explored the population structure and origin of this breed. Furthermore, we applied a genome-wide analysis to detect Arabian-specific genomic regions that display signals characteristic of the action of selection, and therefore may hold variants important for the unique physiological traits of the Arabian horse.

## Results

### Genomic measures of diversity

A total of 378 Arabian horses from twelve countries were included in this study, including populations from Qatar, Iran, UAE, Poland, USA, Egypt, Jordan, Kuwait, United Kingdom, Australia, Denmark, and Canada (Supplemental Fig. S1). We first measured genome-wide heterozygosity within individual horses for 30,398 SNPs, observing ranges from 0.3 for the Saudi Arabian samples to 0.33 for the Iranian samples. Only the Straight Egyptian subgroup differed significantly from the other Arabian types, with an average individual heterozygosity of 0.26 (Supplemental Table S1). Excepting the Straight Egyptian, the Arabian subgroups did not have significantly lower heterozygosity than many of the other modern breeds used for comparison including the Thoroughbred, a breed that has a well-documented narrow base of founding animals^19^.

Regarding genetic differentiation between Arabian subgroups, pairwise *F*_*ST*_ values were found to be significant in all comparisons (permutation p-value <1 × 10^-4^), with highest differentiation observed between the Thoroughbred breed and the Straight Egyptian Arabian subgroup (*F*_*ST*_ = 0.23), while the lowest value was between the multi-origin ancestry and Polish subgroups (*F*_*ST*_ = 0.02) (Supplemental Table S2).

We also conducted an analysis of molecular variance (AMOVA) in Arlequin. In addition to geography-based groups (“Lineage” in Supplemental Table S1), a breed-based grouping was included in the model (population level in AMOVA). The AMOVA results indicated that most variation was within individuals (86%, p < 5 ×10^-5^), with small contributions from variation among breeds (6.7%, p = 0.07), among groups within breeds (5.5%, p < 5 × 10^-5^), and among individuals within groups (1.7%, p = 0.01).

### PCA

**Figure 1:**
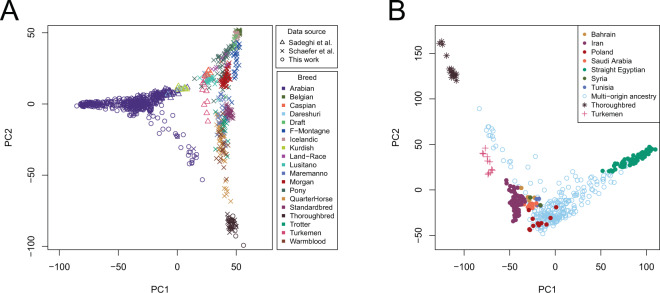
The Arabian is a distinct breed with diverse lineages, having little apparent relationship to the Thoroughbred. Principal component analysis of 378 Arabian horses sampled in this study (**A**) among a reference set including samples from 18 additional global breeds from^13^ and^22^, with symbol shape indicating data source and symbol color indicating breed (data set: 917 samples across 30,967 SNPs); and (**B**) with 71 Persian Arabian and 11 Turkemen samples from^13^, and 17 Thoroughbred samples collected in this study, with symbol shape indicating breed, and symbol color indicating Arabian breed lineage, except for the Thoroughbred and Turkemen groups (data set: 477 samples across 56,239 SNPs). Percent variance explained: (**A**) PC1 = 5.6%, PC2 = 2.2%; (**B**) PC1 = 4.8%, PC2 = 2.5%. PC3 is plotted in Supplemental Figs. S2 and S3. See also Supplemental Fig. S4.

We applied principal component analysis (PCA) to an expanded dataset that included 378 Arabian horses of diverse origin collected for this study, combined with data from Sadeghi *et al*.^13^ and Schaefer *et al*.^22^ for a total of 482 Arabian horses and 435 additional horses representing 18 distinct breeds (analysis data set: 30,967 SNP genotypes from 917 samples) (Fig. 1A). The Arabian horses form a cluster that is clearly separated from most other horse breeds, with the exception of horses of the rare Dareshuri and Kurdish breeds from Iran (Fig. 1A). The Arabian cluster is also broad, particularly in the PC1 dimension, suggesting a high level of genetic diversity within the global Arabian population. The other breed with a similarly broad cluster across the PCA was the American Quarter Horse, a breed notable for its division into several subgroups that also cluster separately in PCA^23^ and differ in the frequency of alleles determining susceptibility to recessive inherited diseases^24^. In our analysis, the Quarter Horse samples displayed diversity across PC2. Finally, the Arabian cluster appears distinct from the Thoroughbred cluster, with the exception of a small number of Arabian horses that will be discussed below.

To investigate relationships among Arabian horses of unique identifiable lineages, we applied PCA to just the Arabian population of 378 samples collected in this study, with the addition of the 71 Persian Arabian samples from Iran^13^, and included Thoroughbred (this study) and Turkemen samples^13^ as out-groups (analysis data set: 56,239 SNP genotypes from 477 samples). In general, the horses from the same lineage tend to cluster together, with the Straight Egyptians separating far from the main cluster (Fig. 1B). As expected, the horses of multi-origin ancestry are scattered across the PCA plot.

**Figure 2:**
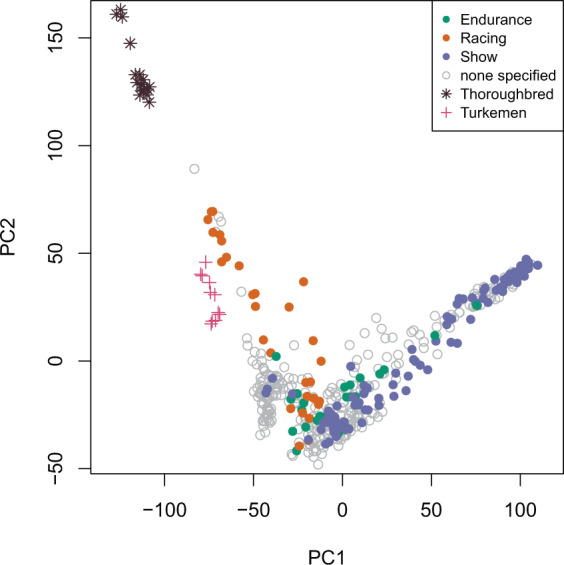
The Arabian breed shows genetic differentiation associated with performance use. Principal components in Fig. 1B were replotted to reflect known competition types for a subset of Arabian horses. Arabian samples were color coded by use (type of competition) in endurance competition, flat course racing or show. Racing horses (orange) plot in the PCA space between the Thoroughbred racehorse and other subgroups of Arabian horse.

Among the samples collected in this study, we identified a subset of Arabian horses with publicly available performance records from participation in competitions. For Arabian horses, the three principal types are show competition (where the horses are judged on conformation and movement), and two mounted sports: long-distance endurance racing (distances of 80-160 km per day), and short distance flat-course racing (1000 m – 3200 m). We reexamined the PCA results in the context of these different types of competitive events. As shown in Fig. 2, most horses of the Straight Egyptian lineage are used only for show competitions. Horses used for endurance races tended to be drawn from the Polish and multi-origin clusters. Finally, many of the Arabian horses used for flat racing were placed in PC space closer to the Thoroughbred cluster (Fig. 2). This relationship may have historical significance and warrants further investigation.

### Structure

**Figure 3:**
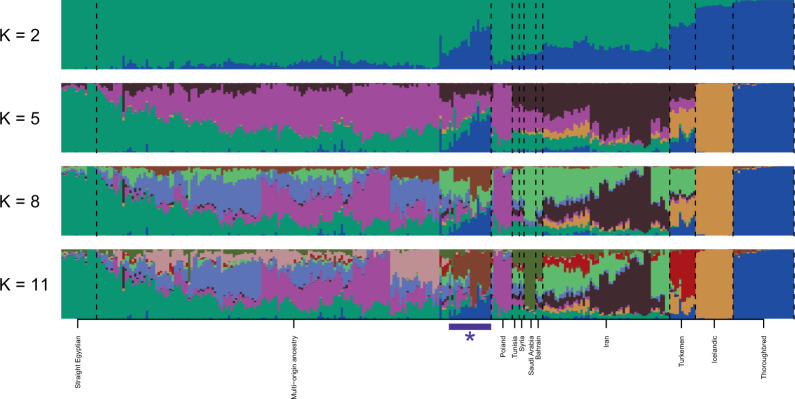
Individual lineages of the Arabian breed display complex ancestry. STRUCTURE cluster assignments are plotted for number of clusters *K* = 2, 5, 8, and 11 (top to bottom panels). Each cluster in a given analysis (panel) is represented by a separate color. The plotted 312 samples represent Arabian breed subgroups as well as Turkemen, Icelandic and Thoroughbred breeds. Sample order is the same in each panel. 47,436 SNPs were included in the analysis. The purple bar and asterisk mark the cluster of multi-origin samples showing shared ancestry with Thoroughbred samples.

We described evidence of global shared ancestry using the STRUCTURE program^25^ within the same collection of horses visualized by PCA in Fig. 1B (after pruning for relatedness [IBD < 0.25] to 296 animals). We included the Icelandic population (16 individuals with IBD < 0.25) from Schaefer *et al*.^22^ as an additional outgroup (analysis data set: 47,436 SNP genotypes from 312 samples). The Thoroughbred breed, a logical comparison based on written history, appears relatively homogeneous within breed, consistent with the closed breeding practices recorded in the Thoroughbred studbook since the late 1700s (Fig. 3). Similarly, the geographically isolated Icelandic breed appears homogeneous and unique at K ≥ 5. In contrast, individual horses from Middle Eastern countries, including Iran, and the large group of horses outside the Middle East and with well-documented recent multi-origin ancestry, show varying degrees of admixture across the K- populations. A subset of multi-origin ancestry horses possesses an unusually large proportion of shared ancestry with the Thoroughbred (Fig. 3, highlighted by the purple bar and asterisk), leading us to further investigate this undocumented admixture.

### RFMix analysis of outcrossing to the Thoroughbred

**Figure 4:**
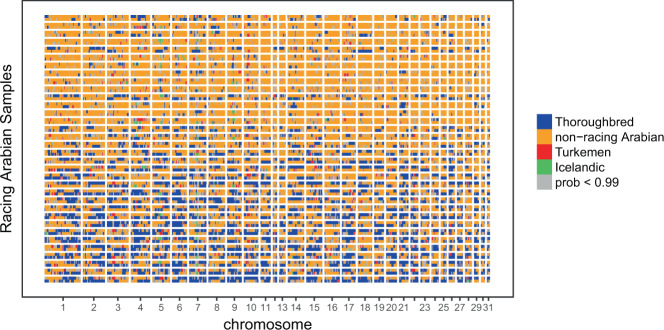
Admixture analysis indicates presence of Thoroughbred ancestry in Arabian horses used for flat course racing. RFMix was applied to the set of 34 “racing Arabian” samples. Segments assigned to one of four reference groups with posterior probability ≥ 0.99 are colored as indicated in the legend. No assignment was made if all posterior probabilities were <0.99 (gray segments). Each row is a haplotype, with individuals (pairs of haplotypes) separated by horizontal white lines. Samples were ordered based on the first principal component (PC1) of the PCA in Fig. 2. See also Supplemental Fig. S5.

We used RFMix^26^ to explore the genetic relationship among Arabian horses used for flat racing competitions and the Thoroughbred breed (analysis data set: 293,293 SNP genotypes from 335 samples). We detected genomic segments of Thoroughbred origin totaling from 2% to 62% of the genome in racing Arabians (Fig. 4). In some registered flat-course racing Arabian horses, near full length chromosomes appear to originate from Thoroughbreds (Fig. 4, and Supplemental Fig. S5). We included data from Icelandic horses as a negative control, as any contact between Icelandic and Arabian horses in the last 1000 years would be extremely unlikely given the recorded history of these two breeds. As predicted, we found negligible evidence for shared ancestry between the Icelandic horse and Arabians (1% overall). We also included samples of Turkemen horses from Iran (including individuals of the Akhal-Teke breed, a descendant of the extinct Turkoman horse) as a reference group, given previous studies that have suggested that the Arabian is closely related to these populations^27^, and the possibility of admixture, given the overlapping historical geographic ranges of these two populations. However, we observed minimal local ancestry assignment to the Turkemen reference group (2% overall). The autosomal admixture patterns illustrated by the RFMix analysis (Fig. 4) strongly indicate that some modern flat-course racing Arabians have recent Thoroughbred ancestry in their heritage.

### Y chromosome haplotyping

**Figure 5:**
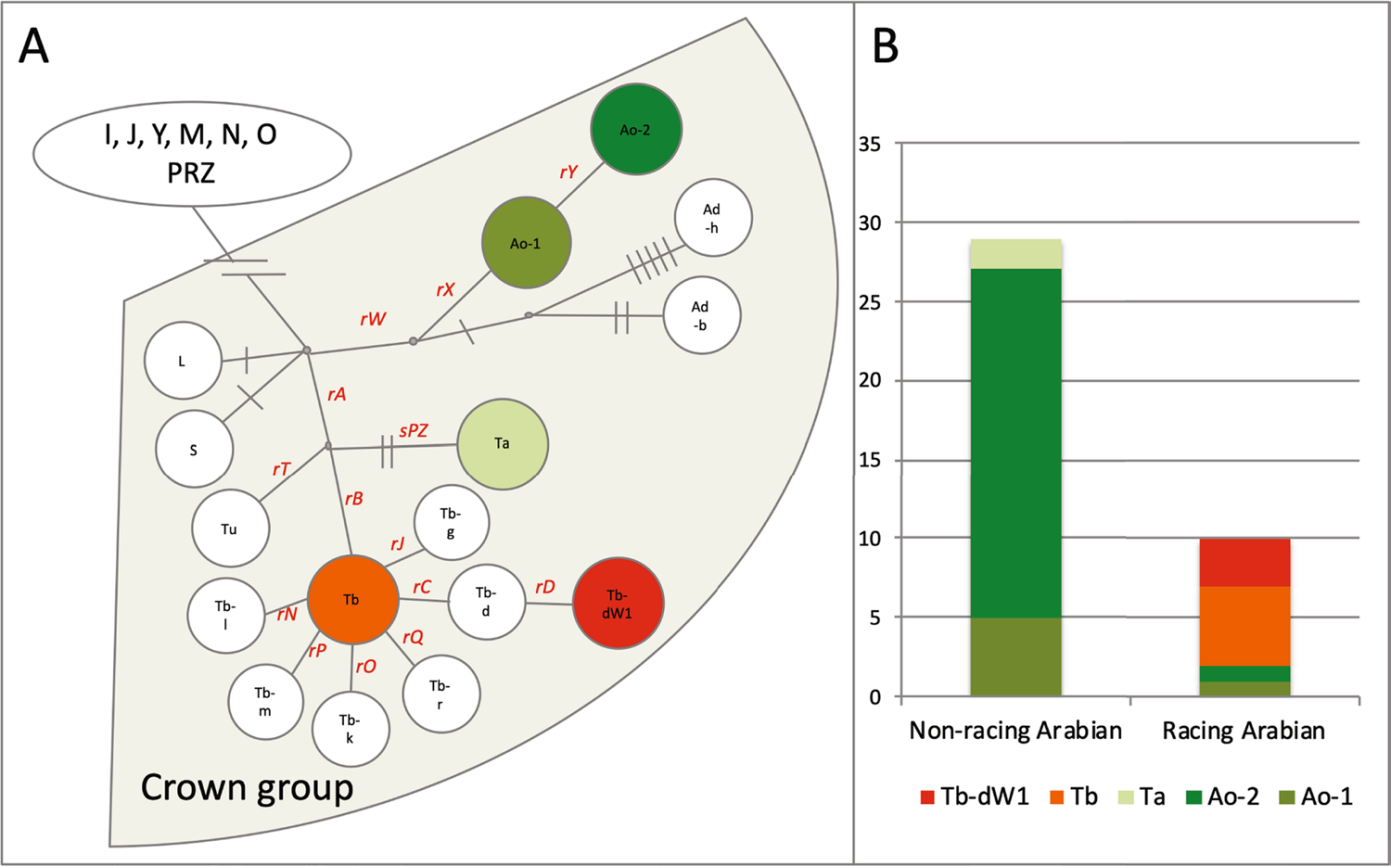
Y chromosome haplotyping supports use of Thoroughbred sire lines in the Racing Arabian subgroup. (**A**) Reduced haplotype network map of the male-specific region of the Y-chromosome according to^21^ with SNPs genotyped in this study in red. Detected haplotypes are colored, and haplotypes in white circles were not detected in the 39 Arabian males genotyped. Haplotypes in green were previously detected in Arabian horses in other studies^21,28^. The ‘Whalebone’ haplotype, Tb-dW1, is colored in red. (**B**) Y haplotype frequencies in racing versus non-racing Arabian horses shown in absolute numbers.

Given that RFMix analysis identified recent admixture of Thoroughbred genomic regions into present-day Arabian horse genomes, we explored the sire-dam direction of interbreeding by examining the Y chromosome haplotypes of selected male Arabian horses used for show or racing competitions, using recently published methods^28^. The 29 non-racing Arabian horses all carried one of three Y haplogroups previously associated with Arabian horses (Fig. 5)^21,28^. The two most common haplogroups, Ao-1 and Ao-2, were previously documented as ancestral to modern Arabian horses^21^. The Ta haplogroup is genetically more similar to haplogroups of modern Thoroughbreds, but is only observed in Arabians^28^. Notably, only two of the 10 male race-use Arabian horses examined carried any of these three Arabian Y haplogroups (Ao-1, Ao-2 and Ta). Five of the race-use horses carried the Tb-oB1* haplogroup attributed to the “Byerley Turk” foundation sire of the Thoroughbred breed^28^. Tb-oB1* is found within a variety of breeds and lineages, including the Turkomen. Therefore, these five horses may carry Y chromosomes derived from ancestors common to both racing Arabians and the Thoroughbred breed^28^. However, the remaining three racing Arabian horses carried the Tb-dW1 haplogroup, also known as the Whalebone haplotype. The mutation leading to Tb-dW1 occurred around 1800^29^, after the establishment of the Thoroughbred studbook, and is linked to the “Whalebone” sire line within the modern Thoroughbred breed^28^. Tb-dW1 is almost fixed in Thoroughbred horses and has not been reported previously in modern Arabians. The presence of Thoroughbred-specific Y chromosome haplogroups among Arabian racehorses indicates that the large chromosomal blocks of Thoroughbred origin detected in flat racing Arabian horses are likely derived, at least in part, from crosses with Thoroughbred stallions that occurred after the emergence of the “Whalebone” haplotype in the 1800s.

### Identification of putative selective sweeps

Arabian horses possess unique physiological traits that result from selection for the diverse activities humans raise and train them for, as well as the challenging natural environments in which these horses thrive. We examined genetic divergence as evidence of putative selective sweeps among groups selectively bred for flat-course racing, as well as across subpopulations from diverse geographic and cultural origins. We computed three test statistics (*H*, *H*_*12*_, and Tajima’s *D*) to ask whether the patterns of genetic differentiation across these populations were consistent with neutrality, or whether instead they reflect some operation of directional selection.

**Figure 6:**
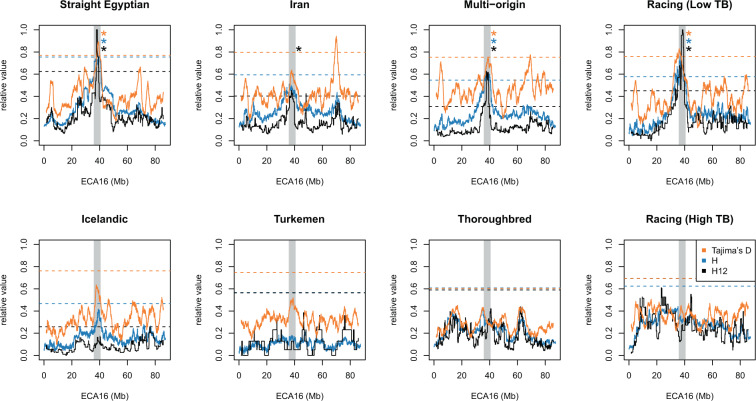
Selection scan statistics highlight a candidate region on ECA 16 containing *GPX1*, a gene important for protection from exercise-induced oxidative damage across Arabian lineages. *H, H*_*12*_, and reflected Tajima’s *D* statistics were all scaled to 0 – 1. 99^th^ percentile threshold values are plotted as dashed horizontal lines for each statistic. The selection scan peak with the highest count of overlapping peaks (“multi-strain” group, *H* statistic, overlapping with 11 other peaks) is shown as the vertical gray region. Colored asterisks indicate which test statistics had peaks exceeding the indicated threshold within the region of interest.

Across all subgroups of the Arabian horse (excepting the racing animals with a high proportion of Thoroughbred admixture), and for all three statistics indicative of genomic regions impacted by selective sweeps (*H*, *H*_*12*_, and Tajima’s *D*), we noted a significant region on ECA 16 specifically not identified in the Thoroughbred, Turkemen and Icelandic control populations (Fig. 6). The region defined by the intersection of six identified peaks, chr16: 37,646,236 – 37,810,203, overlapped just seven Ensembl Gene Models and/or homologous human proteins (*DAG1, NICN1, AMT, TCTA, RHOA, GPX1* and *USP4*). Notably, *GPX1* encodes the glutathione peroxidase enzyme, a selenoprotein that mitigates oxidative damage and is frequently associated with exercise in both human and equine athletes^30,31^. No polymorphism in coding sequences within this region was found (relative to the reference genome assembly from a Thoroughbred horse) among the eight Arabian genome sequences examined for this work.

**Figure 7:**
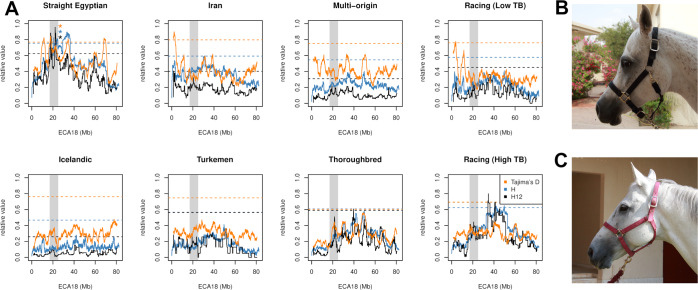
Selection scan statistics reveal a putative selective sweep unique to the Straight Egyptian subgroup on ECA 18. (**A**) *H, H*_*12*_, and reflected Tajima’s *D* statistics were all scaled to 0 – 1. 99^th^ percentile threshold values are plotted as dashed horizontal lines for each statistic. The vertical gray region indicates the genomic region encompassing all 6 peaks observed for the Straight Egyptian group. Colored asterisks indicate which test statistics had peaks exceeding the indicated threshold within the region of interest. (**B**) Representative profile of a Straight Egyptian Arabian Horse and (**C**) of an Arabian horse used for Racing and with no Straight Egyptian ancestry.

The Straight Egyptian subgroup, prized primarily for its beauty and value in the show ring, possesses six putative sweep signals capturing the region chr18:17,001,143-24,991,147 (Fig. 7A), overlapping with 13 annotated genes (Supplemental File S1). One strongly supported peak window, chr18:17,492,252-18,727,012, overlaps just three annotated genes. Of particular interest among these, *TMEM163* encodes a transmembrane protein with a putative function as a zinc transporter^32^. Human GWA studies correlated markers at *TMEM163* with the width of the face between the eyes, and the relative height of the eyes on the face^33^. This human phenotype echoes a key characteristic found primarily in the “Straight Egyptian” subgroup of the Arabian: a markedly concave nasal bone and domed forehead with large eyes (Fig. 7B)^34^.

## Discussion

The Arabian horse presents a paradox within equestrian culture. To those who admire the breed, the gracefully shaped head with dished forehead and wide-set eyes are the iconic representation of the Arabian horse. Furthermore, virtually every horse fancier can recite the story of the influence of Arabian stallions in founding the modern Thoroughbred breed^20,35,36^. To its detractors, the Arabian represents an overly inbred horse breed with a high incidence of inherited autosomal recessive diseases^37^. Here we tested a large collection of 378 Arabian horses from many countries and breeding lines using a combination of whole-genome sequencing and the 670k equine single nucleotide polymorphism (SNP) array. We analyzed this dataset with comparable data in the public domain from 18 other breeds.

Our results challenge long-held assumptions about the Arabian horse. Despite having been dispersed widely across the globe by humans, the breed as a whole maintains a unique genetic identity observable by geometric segregation in a very broad swath of PC space (Fig. 1A). Across Arabians AMOVA revealed a majority of variation captured across individuals, rather than by subgroup. Yet, we observed significant differentiation between all Arabian subgroups examined by *F*_*ST*_ (permutation p-value <1 ×10^-4^). Three subgroups of Arabian horses also segregate uniquely by PCA: the Straight Egyptian, the Polish Arabians, and the horses from Saudi Arabia. This finding agrees with the written histories of these groups, characterized by closely controlled breeding of these lineages over the past 200 years^6^. A similar sub-breed structure has been described in the American Quarter Horse, a registry notable for its functional division into several subgroups that also cluster separately in PC space^23^.

Within the Arabian breed we found evidence of relatively high inbreeding within individuals, especially those belonging to the Straight Egyptian sub-group (mean *F* = 0.30) (Table S1). The Straight Egyptian subgroup also separates from other Arabian types in PCA (Fig. 1B) and by STRUCTURE (Fig. 3). The Straight Egyptian lineage represents only about 3-5% of all Arabians, but it is highly prized by owners who compete in non-ridden horse show competitions. This subgroup may be subject to relatively intense selection for the specific conformation types preferred in the show ring. Despite relatively diverse pedigrees, the high inbreeding values were also observed within individual horses of the multi-origin category. This may reflect population bottlenecks that occurred during exportation of these horses from the Middle East to individual stud farms in the USA and Europe, followed by modern breeding practices that are often driven by a popular sire effect. Inbreeding within some lines of Arabians has increased the occurrence of a number of recessive inherited diseases^38–40^. Similarly, within the American Quarter horse subgroups inbreeding values vary considerably, and the individual subgroups display different associations with inherited diseases^24^.

We identified registered Arabian horses resident in the Middle East that clustered with the Arabian breed, but that carried expanded genetic and phenotypic diversity. In particular, the Syrian/Tunisia/Bahrain and Iranian subgroups examined here displayed complex ancestry (Fig. 3). Such “desert-bred” Arabians often lack deep written pedigrees and have a diversity of physical characteristics that is typical of a landrace, yet these horses still cluster with other modern Arabians by PCA (Fig. 1A), and pairwise *F*_*ST*_ (Table S2). Increased diversity in these subgroups is consistent with a Middle Eastern origin for the modern Arabian horse. This possibility, too, has been suggested by Głażewska^41^, based upon mtDNA genetic studies. Unfortunately, documentable ancestral populations may no longer exist for the Arabian, as for most domesticated horses, and populations of unregistered horses are difficult to identify and sample in this region.

Our results confirm and extend findings reported earlier by two groups. Khanshour and colleagues tested over 600 Arabian horses from several locations in the Middle East, Europe, and the USA^11^. Although they used only 15 polymorphic microsatellites for their study, they identified higher genetic diversity in Arabian horses from the Middle East region than in horses from other regions. Furthermore, they showed evidence for complex ancestry in many Middle Eastern populations, but not in the Straight Egyptian horses. Almarzook and colleagues used the same 670k SNP array that we used in our study, but they tested only 48 horses drawn from Syria and the USA^12^. Even with this small sample size, they observed high levels of genetic diversity in the Syrian horses. They also failed to show correlation between genetic ancestry defined using SNP array data and the traditional definition of maternal strains maintained by Arabian horse breeders, including those in Syria. Neither study examined the diversity of the Arabian horse within the context of a global sampling of horse breeds. However, considered with the findings we present here, these three reports make a strong case for the existence of robust, genetically diverse populations of Arabian horses in the Middle East today.

Thus, although the global population of Arabian horses is diverse, loss of diversity within some subgroups like the Straight Egyptian may be reaching levels sufficient to impact animal health^42^. Indeed at least three recessive genetic diseases segregate within the Arabian horse, and are already of particular concern within some subgroups^9,10^. This finding highlights the need for use of genomic tools to manage inbreeding within these populations as pedigree-based calculations may not accurately measure loss of diversity due to historic events^37^. Likewise, identity-by-state guided breeding decisions could assist in maintaining rare alleles and heterozygosity in endangered populations, both in the Middle East and abroad.

Historical evidence suggests that the Arabian was selectively bred for its distinctive conformation, characterized by a dished facial profile, graceful upright neck, and high tail carriage, over the last 3,000 years^2^. Human-directed selection for phenotypes including athletic endurance and iconic conformational traits like a dished facial profile has likely left “signatures” in the patterns of nucleotide diversity that are detectable in the genomes of modern Arabians. Natural selection imposed by the hot and arid environments under which the Arabian horse was traditionally reared may have also altered regions of the genome, thus conferring a cost to relative fitness. A putative sweep on ECA16 was common to all Arabian subgroups, but not found in control breeds tested here (Fig. 6). Dietary supplementation with selenium is suggested as a method to increase activity of the *GPX1* enzyme (glutathione peroxidase), a candidate gene within this ECA 16 sweep. This increase in activity may reduce oxidative damage to muscle and blood tissues resulting from exercise, thereby improving performance^43^. Many studies have examined changes in blood glutathione peroxidase activity pre- and post- training, and resting blood activity of this enzyme is higher in race-trained Thoroughbreds than in other athletic horses^44^. However, glutathione peroxidase expression or basal activity has not yet been compared across breeds, nor specifically in athletic types vs. non-athletic types of horse. Therefore, conserved regulatory motifs and additional sequencing across breeds with diverse athletic ability may be logical targets of further investigation across *GPX1*, as well as other genes within this candidate sweep region.

We also observed evidence of selective breeding for specialized competitions such as racing and show across Arabian subgroups (Fig. 2). We identified one such putative selection signature specific to the Straight Egyptian horse on ECA18 (Fig. 7). Although future work is needed to better determine any phenotypes attributable to this sweep, the Straight Egyptian does possess a particularly notable expression of the highly prized concave Arabian facial profile. Thus, the Arabian horse may be an advantageous model for identification of genes contributing to skull morphology, as has been previously demonstrated in the dog^45^.

Finally, we identified undocumented relationships between the Thoroughbred breed and the modern Arabian that are contrary to breed registry regulations and dispute long-held myths. Although celebrated in many historical accounts^20,36^, the three “Arabian” sires recorded as the main male founders of the Thoroughbred breed (the “Darley Arabian”, “Godolphin Arabian” and “Byerley Turk”) were likely individuals of other Oriental horse populations, and the Arabian breed appears to have contributed little to the autosomal genomic content of the modern Thoroughbred (Fig. 3). This disagreement may stem from a simple confusion surrounding the naming of these horses. For example, the “Darley Arabian” was certainly a stallion purchased by Thomas Darley and shipped from within Arabia, but its breed was likely of yet unknown genetic origin^46^. The possibility that “Arabian” stallions that contributed to the founding of the Thoroughbred were from a population unrelated to most Arabian horses was discussed 20 years ago by the distinguished American horseman, Alexander Mackay-Smith in a book written for enthusiasts of the Thoroughbred racehorse^47^, and idiosyncrosies in nomenclature regarding the origin of these stallions were noted as early as 1893^48^.

In contrast, we detected evidence of modern outcrossing of registered racing-type Arabians to the Thoroughbred, a practice that is prohibited by Arabian horse registries (Figs. 2–4). This finding was confirmed by examination of Y-chromosome haplotypes, where several racing Arabians possessed the “Whalebone” haplotype specific to modern Thoroughbred lineages^21^ (Fig. 5).

Taken together, our observations lead us to hypothesize that only a small proportion of total genetic diversity left the Middle East when Arabian horses were imported to Europe and the USA over the past 200 years. The residual genomic and phenotypic diversity within the Arabian horse breed in the Middle East is indicative of a high long-term effective population size, and also reflects the overall robust genetic health of this population. The genetic history of the Arabian thus holds greater interest and fascination than the myths that have surrounded this charismatic breed of horse for over 200 years. The application of modern breeding techniques, such as artificial insemination, is producing an international pedigree of modern Arabian horses marked by genetic homogenization, and in some cases, severe inbreeding and pedigree errors. This emphasizes the critical need for more detailed studies of genomic diversity in native Arabian horses in order to enable conservation efforts and manage inbreeding in at-risk subgroups. The Middle Eastern subgroups examined here (Iranian, Bahraini, Tunisian, Syrian and others) may represent refugia of genetic diversity crucial to the future of the Arabian horse.

## Methods

### Samples and populations

Sample counts per breed and per lineage group are reported in Supplemental Table S3. The geographical location of the collected samples is shown in Supplemental Fig. S1. Blood and hair sampling from each horse was completed with the consent of the horse owner under all appropriate institutional guidelines and was approved by the Animal Care and Use Committees at Cornell University (2008-0121 and 2013-0057) and the University of Florida (201408459 and 201708411). Genomic DNA was extracted using conventional methods. Although most modern Arabian horse registries fall under the umbrella of the World Arabian Horse Organization (WAHO) and are registerable across the various national breed organizations, some Arabian horse populations, like those in Iran (Persian Asil horse), have remained relatively geographically and genetically distinct. The Persian Arabian studbook extends back only to 1952, but it is likely that the lineages may be more ancient. In Iran, Mary Gharagozlou worked tirelessly during the second half of the 20^th^ century to document the pedigrees of pure-bred Persian Arabian horses, including lineages tested in this study^49^. We sought out horses that were described by their ownership history and pedigree as representing examples of diverse locally bred lines. Our samples included horses with recorded ancestry tracing back to several countries in the Middle East and North Africa: Bahrain, Iran, Saudi Arabia, Syria, Tunisia and horses from Poland.

We grouped Arabian horse populations based on the origin recorded for founders identified in pedigrees maintained by the studbook. The “Straight Egyptian Arabian” horses are those that can trace some ancestry back to the horses bred by the Bedouin tribes of Arabia. These horses are often associated with the Egyptian stud farm of Abbas Pasha or the Egyptian Royal Agricultural Society breeding programs^2^. Outside of Egypt, records on these horses are currently maintained by the Pyramid Society organization, and in North America the Al Khamsa Arabian registry. The Straight Egyptian group includes samples of horses residing today in many different countries including USA, Kuwait, UAE, Denmark, Egypt, and Qatar.

Poland has a long history of selective breeding of Arabian horses^6,8^. Arabian lineages were first imported to Poland in 1778 and their numbers supplemented over the years by new horses imported directly from the Middle East beginning in 1803. Violence during World War I and II nearly destroyed all Polish-Arabian horses and the modern lines maintained in the Polish breeding program were started between 1810 and 1902^15^.

We also identified groups of samples with origins in Bahrain, Iran, Poland, Saudi Arabia, Syria, and Tunisia, as well as a collection of 17 Thoroughbred and two Standardbred samples as reference outgroups. Seven registered Akhal-teke horses and four horses of the closely related Yamut breed of horse were grouped together under the label “Turkemen”, as done by Sadeghi et al.^13^. Finally, there remained a large group of horses of mixed lineage and origin, labeled as “multi-origin ancestry”. Many of these horses were sampled in the United States and Europe, where over the last ~150 years Arabian horse breeders utilized bloodstock obtained from different origins imported at various times.

### Genotyping and SNP filtering

Genotyping was conducted at GeneSeek Inc. (Lincoln, NE, USA) and Affymetrix Inc. (Santa Clara, CA, USA) using the Axiom Equine Genotyping Array, a chip-based SNP array with 670, 796 K SNPs. Raw CEL files were loaded into the Axiom Analysis Suite for SNP calling. Sample filters were set to DQC ≥ 0.82 and call rate ≥ 90, whereas SNP filters were set to the recommended diploid values. After genotyping, the variant list was restricted to PolyHighResolution clusters with a FLD ≥ 3.6.

For whole genome sequencing data, raw Illumina reads were first processed with trimmomatic v0.33 using the options ILLUMINACLIP:TrueSeq. 3-PE.fa:2:30:10 LEADING:3 TRAILING:3 SLIDINGWINDOW:4:20 MINLEN:60^50^. Variants were called following the GATK Best Practices pipeline. First, processed reads were aligned to equCab2 using BWA mem v0.7.15 with default parameters, either as paired or single for reads that lost their pair to filtering^51^. Read alignments were further processed in Picard using the CleanSam and SortSam modules, paired and single end reads were merged with the MergeSamFiles module, and then were finally processed with the MarkDuplicates, AddOrReplaceReadGroups, and BuildBamIndex modules (http://broadinstitute.github.io/picard/). GATK Queue v.3.7.0 was used to run BaseRecalibration and HaplotypeCaller on each individual, using variants generated from iterative variant calling on six horse genomes^52,53^. Individual GVCF files were merged with CombineGVCFs and were genotyped with GenotypeGVCFs using the options --includeNonVariantSites and -L affymetrix.intervals to generate genotypes at all genotyping chip locations.

Genotype calls from each genotyping array batch and the whole genome sequences were combined sequentially using PLINK v. 1.90^54^. First, Affymetrix and GeneSeek calls were merged using PLINK with filters set to 90% SNP genotyping rate and 1% minor allele frequency. A subset of multi-origin ancestry Arabians (from mixed origins) were used to test all SNPs for Hardy Weinberg Equilibrium. Autosomal variants with *P*-values <0.005 (corrected for multiple testing) were removed from the genotype files. SNPs derived from whole genome sequencing were then merged to generate the final set of variant calls for downstream analysis, removing any variant with lower than 80% genotyping rate, 1% minor allele frequency, or flagged as multi-allelic. Finally, samples with genotyping rate <95% were removed. After applying these filters, the data set included 343,367 SNPs.

Further filtering was conducted using PLINK v.1.9 based on minor allele frequency, SNP genotyping rates, and pruning of SNPs in linkage disequilibrium (LD). We applied the following filters for specific analyses: PCA and Arlequin: --maf 0.02 --geno 0 --indep-pairwise 50 5 0.5; STRUCTURE: --maf 0.02 --geno 0 --indep-pairwise 50 5 0.3; phasing with shapeit (for RFMix and selection scans): --maf 0.01 --geno 0.02 (no LD pruning).

Given the presence of closely related individuals within subgroups of horse samples gathered for this study, we filtered individuals by relatedness to provide less biased sets for use in some of the analyses. We filtered samples based on pairwise identity-by-descent (IBD) and inbreeding coefficients. IBD values were calculated using the --genome option in PLINK, and we limited the non-related sample sets to pairwise IBD < 0.25. We used the “Fhat3” inbreeding coefficient output by PLINK using the --ibc option and excluded samples with Fhat3 > 0.257 (threshold = mean + 2*stdev).

### Expanded data set

In order to look at the samples collected in this study in the context of many different horse breeds, we merged our data set with data from two studies: 101 670 K Equine SNP chip samples from^13^ (71 Iran Arabian samples and 30 samples from four different Iranian horse breeds) (“Sadeghi et al.” data set); and 419 samples from the multi-breed data set from^22^, including 314 samples that were genotyped with the 670 K Equine SNP chip and 105 samples with whole genome sequencing data (“Schaefer et al.” data set). Sample counts per breed and per source study are shown in Supplemental Table S4. We used the --merge option in PLINK to combine the data sets. The final expanded data set included genotype data for 917 samples from 19 different horse breeds across 319,353 SNPs. We did not observe evidence of study-specific batch effects (Fig. 1A), nor platform-specific batch effects (Fig. S6).

### Diversity, inbreeding, and molecular variance analysis

Due to the small sample sizes for the Bahrain, Syria, and Tunisia groups, and given we observed similarity in these samples on the PCA plot (Fig. 1B) and in the STRUCTURE analysis (Fig. 3), we combined these groups into a single group for population diversity analyses. Expected heterozygosity (*H*_*E*_), mean observed heterozygosity (*H*_*O*_), and method-of-moments *F* inbreeding coefficient estimates were calculated in PLINK using–het, and pair-wise differences between groups were assessed by a Tukey-Kramer test, given unequal variances revealed by the Levene’s test (JMPv14.1.0, SAS Inc.). PGDSpider version 2.0.8.2 was used to convert PLINK files to Arlequin format^55^. Arlequin version 3.5.2.2 was used to calculate mean pairwise population differentiation (*F*_*ST*_), and to conduct an analysis of molecular variance (AMOVA)^56^. Significance testing was conducted for *F*_*ST*_ and AMOVA results in Arlequin using 20,000 permutations. In the AMOVA, populations were defined based on breed, and groups were defined based on Arabian lineage (or breed, in the case of the Thoroughbred and Turkemen samples).

### Population structure

Principal Component Analysis (PCA) was conducted in R^57^ using the function prcomp() with scale = TRUE (variables scaled before the analysis). To investigate possible influence of over-representation of our Arabian cohort on the PCA result, we performed several additional PCA runs using different subsets of Arabian horses selected using varying criteria. The PCA plots that included balanced numbers of horses across breeds, and across subgroups within the Arabian breed, had very similar shapes and distributions of clusters (Supplemental Fig. S4).

We applied the software STRUCTURE in order to cluster the samples and investigate genetic structure in the data set^25^. In addition to the samples generated in this study, we added the Thoroughbred and Icelandic samples from^34^, in order to increase Thoroughbred sample size and as an additional outgroup, respectively. Following previous findings that closely related samples can confound STRUCTURE results^58^, we filtered out closely related samples as described above (require pairwise IBD < 0.25). We ran STRUCTURE for number of clusters (*K*) ranging from 1 to 14. We set BURNIN = 30,000 and NUMREPS = 50,000 and ran 5 replicates for each value of *K*.

In order to determine the optimal *K*, we considered several approaches, including the *K* yielding maximum mean estimated lnP(D), and Evanno’s Δ*K*^59^, as implemented in Structure Harvester^60^. CLUMPP was applied to combine replicate runs using the LargeK Greedy algorithm^61^. The optimal number of clusters K was evaluated using the plots in Supplemental Fig. S7. Evanno’s Δ*K* method suggested optimal *K* = 2, with smaller peaks at *K* = 5 and *K* = 11. Maximum mean estimated log probability of the data (lnP(D)) was observed at *K* = 11, and visual inspection of mean lnP(D) across *K* = 1 to 14 showed an “elbow” at *K* = 5. Accordingly, we plotted STRUCTURE results for *K* = 2, 5, 8, 11 in Fig. 3.

STRUCTURE results were plotted in R. Plots were generated for a defined set of selected *K* values. Similar clusters in sequential *K* results were identified, and colors were kept consistent for these similar clusters across plots for different *K* values. Samples were ordered within each group by hierarchical clustering of the STRUCTURE results at *K* = 11. The clusters of samples in the multi-origin ancestry group were ordered based on the fraction of each cluster assigned to the Straight Egyptian and Thoroughbred groups.

### Local ancestry inference

Local ancestry inference requires phased haplotype data as input. We used SHAPEIT v2.r837 to phase the genotype data for each autosomal chromosome^62^. We ran SHAPEIT with 200 conditioning states (--states) and an estimated effective population size (--effective-size) of 1,000. We also used the --duohmm option in order to take advantage of known parent-offspring pairs in the data set and set window size (-W) to 5 Mb, as suggested in the SHAPEIT documentation.

Local ancestry inference was conducted using RFMix^26^. A set of flat racing Arabian samples was identified as the putatively admixed group. This “Racing Arabian” group (*N* = 34) included all horses known to compete in flat racing, as well as five additional samples with unknown performance records that clustered with the flat racing samples in PCA (Fig. 2; five Arabian samples with PC1 < -40 and PC2 > 20). We included four reference groups in the RFMix analysis: Thoroughbred (*N* = 41; samples from both this study and from^22^), non-racing Arabians (*N* = 231), Turkemen (*N* = 11; samples from^13^), and Icelandic (*N* = 18; samples from^22^). The non-racing Arabian reference group included all Arabian samples collected in this study that were not already included in the putatively admixed group and also passed the relatedness filters described above (pairwise IBD < 0.25). The relatedness filter was applied in order to reduce the sample size of the non-racing Arabian reference group, given the smaller size of the other reference groups included in the analysis. The final phased data set input to RFMix included 335 samples and 293,293 SNPs.

We ran RFMix with the PopPhased option, minimum node size (-n) of 5, and generations since admixture (-G) set to 9. Local ancestry assignments to segments of the “Racing Arabian” sample genotypes were only made when posterior probability ≥ 0.99 for one of the reference groups. Otherwise, no assignment was made. Results were plotted in R, with the “Racing Arabian” samples ordered based on values of the first principal component (PC1) in Fig. 2.

### Y-chromosome haplotyping

Y-chromosome haplotypes were determined by genotyping 14 key Y-chromosomal variants using LGC KASP® technology. Variant information, including coordinates and flanking regions, is given in^28^. Haplotypes and their phylogenetic relationship were reconstructed as described in^21^.

Because the Y-haplotyping procedure requires both higher quality and a larger quantity of DNA, we were only able to perform Y-haplotype analysis for five of the 12 male racing Arabian samples included in the RFMix analysis (Fig. 4), and 11 of the male non-racing Arabian samples. In order to strengthen the analysis, we conducted the Y-haplotyping analysis for new samples from five male racing Arabians and 18 male non-racing Arabians.

### Signatures of selection

Aggregation of several alternative methods to detect selection signatures has been suggested as a way of increasing the reliability of selection signature studies^63^. Here, we computed *H*, *H*_*12*_, and Tajima’s *D* statistics. The *H* statistic was estimated using the program H-SCAN, downloaded from: https://messerlab.org/resources/ (April 2015), which measures the average length of pairwise haplotype homozygosity tracts around a given genomic position in base pairs^64^. *H* values were estimated at each SNP position in the data set. All scans were run using default H-scan parameters. *H*_*12*_ and Tajima’s D values were quantified over windows of a fixed number of SNPs on our genotyping chip (351 SNPs). In order to achieve a more uniform SNP density across the genome for these window-based scans, high density regions (>16 SNPs per 0.1 Mb) were pruned to 13 SNPs per 0.1 Mb before conducting the analysis. The estimated values of each statistic were then given at the position of the center SNP of the window. *H*_*12*_ values were estimated following the definition provided in^65^. Tajima’s *D* values were variance-normalized according to the formulas given in^66^. In evaluating resulting candidate regions under selection, we excluded broad regions (>5 Mb) where the selection signal is less specific. We noted large peaks near the centromere of ECA 7 and 11 in many subgroups. As these were also noted by Petersen *et al*.^67^, and were not specific to any single subgroup, these features may be due to a structural alteration in the pattern of LD in these regions and were not considered relevant to the Arabian horse specifically.

Selection scans were applied to 8 different sample groups. We split the 34 “Racing Arabian” samples from the RFMix analysis into two groups of 17 samples each based on higher/lower proportion Thoroughbred-assigned local ancestry. We also included origin-based groups for the three origin groups with sufficient sample size: Iran, Straight Egyptian, and Multi-origin Ancestry. Samples with known performance use were excluded from these origin-based groups in order to avoid potentially confounding use-specific signals. Finally, we included the same three out-groups used in the RFMix analysis (Icelandic, Thoroughbred, and Turkemen) (Supplemental Table S5).

The significant genomic regions revealed by *H*, *H*_*12*_, and Tajima’s *D* were identified using a 99^th^ percentile threshold cut-off and lists of genes in these regions were then determined using BioMart tool at Ensembl (www.ensembl.org/biomart) and the horse reference genome assembly EquCab2 (http://asia.ensembl.org/Equus_caballus/Info/Index). A full list of identified regions is reported in Supplemental File S1.

## Supplementary information

Supplementary Information.

Supplementary Information.

## Data Availability

The genotype data generated during this study are available at Mendeley Data: 10.17632/mkk5khxrbp.3. All raw sequencing data generated in this study has been submitted to the ENA under study #PRJEB33818.
