# Supplementary material for: Genome Diversity and the Origin of the Arabian Horse
Source: Sci Rep. 2020 Jun 16;10:9702. doi: 10.1038/s41598-020-66232-1 (PMC7298027; doi:10.1038/s41598-020-66232-1)
Supplement: Supplementary file 2 — Supplementary Information. [file 41598_2020_66232_MOESM2_ESM.pdf]

Genome Diversity and the Origin of the Arabian Horse

Supplemental Information

Elissa J. Cosgrove*[1], Raheleh Sadeghi*[2,4], Florencia Schlamp[1], Heather M. Holl[3], Mohammad Moradi-Shahrbabak[4], Seyed Reza Miraei-Ashtiani[4], Salma Abdalla[5], Ben Shykind[5,6†], Mats Troedsson[7], Monika Stefaniuk-Szmukier[8], Anil Prabhu[9], Stefania Bucca[10], Monika Bugno-Poniewierska[8], Barbara Wallner[11], Joel Malek[12], Donald C. Miller[2], Andrew G. Clark[1], Douglas F. Antczak[2], Samantha A. Brooks[3‡]

* These authors contributed equally.
†Current Affiliation
‡Corresponding Author: Samantha.brooks@ufl.edu

1- Department of Molecular Biology and Genetics; Cornell University; Ithaca, NY, 14853; USA
2- Baker Institute for Animal Health, College of Veterinary Medicine; Cornell University; Ithaca, NY, 14853; USA
3- Department of Animal Science, UF Genetics Institute; University of Florida; Gainesville, FL, 32610; USA
4- Department of Animal Science, College of Agriculture and Natural Resources; University of Tehran; Karaj; Iran
5- Department of Cell and Developmental Biology and Biochemistry, Weill Cornell Medical College in Qatar; Doha; Qatar
6- current address: Prevail Therapeutics; New York, New York 10016; USA
7- Department of Veterinary Science, Maxwell H. Gluck Equine Research Center; University of Kentucky; Lexington, KY, 40546, USA
8- Department of Animal Reproduction, Anatomy and Genomics; University of Agriculture in Kraków; Kraków, Poland
9- The Hong Kong Jockey Club, Hong Kong, Central and Western, China
10- Reproduction/Theriogenology, Equine Veterinary Medical Center; Doha; Qatar
11- Institute of Animal Breeding and Genetics, University of Veterinary Medicine Vienna, Vienna 1210, Austria
12- Department of Genetic Medicine; Weill Cornell Medical College in Qatar; Doha; Qatar

RUNNING TITLE: Genome Diversity in the Arabian Horse

KEYWORDS: genetic diversity, population structure, Arabian horse, breed history, selection

Contents

Figure S1. Related to Methods. Geographic location of the samples collected in this study.

Table S1. Related to Results. Summary of classical population genetic metrics.

Table S2. Related to Results. Pairwise FST values.

Figure S2. Related to Figure 1A. Principal Component Analysis (PCA) PC3 plots for Figure 1A.

Figure S3. Related to Figure 1B. Principal Component Analysis (PCA) PC3 plots for Figure 1B.

Figure S4. Related to Figure 1A. Principal component analysis (PCA) for subsets of the expanded data set.

Figure S5: Related to Figure 4. Distribution of RFMix Thoroughbred-assigned local ancestry block lengths.

Table S3. Related to Methods. Counts of samples collected in this study.

Table S4: Related to Methods. Counts of samples included in expanded data set.

Figure S6: Related to Methods. Principal Component Analysis (PCA) plots for merged data set (Figure 1A) labeled by platform.

Figure S7: Related to Methods and Figure 3. STRUCTURE results used to select optimal number of clusters K.

Table S5. Related to Methods and Figures 6 and 7. Sample groups used in selection scans.

File S1. Related to Figures 6 and 7. Table of all peaks identified in selection scans.

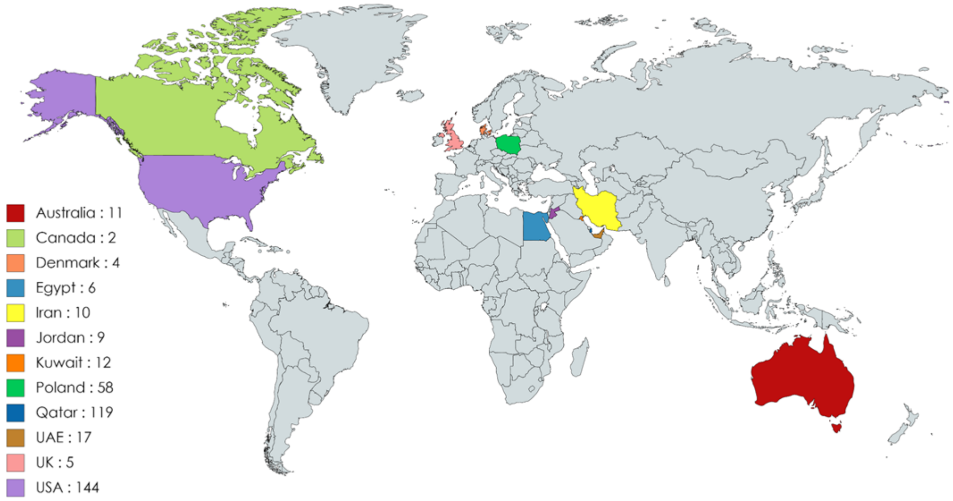

Figure **S1**: **Related to Methods. Geographic location of the samples collected in this study.**

Table **S1**: **Related to Results. Summary of classical population genetic metrics.**

Breed	Lineage	Samples	Observed Mean Het (SD)	Mean F (SD)
Arabian	Bahrain/Syria/Tunisia	10	0.31 (0.01)	0.17 (0.03)[bc]
	Iran	80	0.33 (0.03)	0.12 (0.08)[cd]
	Multi-origin ancestry	259	0.32 (0.02)	0.14 (0.05)[bc]
	Poland	11	0.32 (0.01)	0.14 (0.04)[bcde]
	Saudi Arabia	12	0.30 (0.04)	0.20 (0.10)[b]
	Straight Egyptian	77	0.26 (0.02)	0.30 (0.05)[a]
	(Schaefer et al.)	33	0.32 (0.03)	0.14 (0.07)[bc]
Belgian		21	0.33 (0.02)	0.13 (0.06)[bcde]
Caspian		7	0.35 (0.03)	0.07 (0.09)[cdefgh]
Dareshuri		5	0.35 (0.01)	0.06 (0.02)[cdefgh]
Draft		8	0.33 (0.01)	0.11 (0.03)[bcdefgh]
F-Montagne		29	0.34 (0.01)	0.08 (0.04)[defgh]
Icelandic		18	0.32 (0.01)	0.13 (0.04)[bcde]
Kurdish		7	0.36 (<0.01)	0.04 (0.01)[defgh]
Land-Race		7	0.32 (0.05)	0.14 (0.13)[bcde]
Lusitano		21	0.33 (0.02)	0.11 (0.06)[cdeh]
Maremanno		24	0.36 (0.01)	0.05 (0.03)[fg]
Morgan		61	0.34 (0.02)	0.09 (0.07)[defh]
Pony		46	0.34 (0.02)	0.07 (0.04)[efgh]
QuarterHorse		51	0.35 (0.02)	0.05 (0.05)[fg]
Standardbred		41	0.32 (0.03)	0.14 (0.07)[bc]
Thoroughbred		41	0.32 (0.01)	0.14 (0.04)[bc]
Trotter		14	0.35 (0.02)	0.06 (0.05)[defgh]
Turkemen		11	0.36 (0.01)	0.03 (0.01)[fgh]
Warmblood		23	0.36 (0.01)	0.03 (0.03)[g]

Analysis was conducted using PLINK v.1.9. The data set included 917 samples and 30,398 SNPs (same filtered data set as Figure 1A, excluding the X chromosome variants). Expected heterozygosity was 0.37. Mean F values with the same superscript letter were not significantly different by a Tukey-Kramer test ($p < 0.05$). Het: Heterozygosity; SD: Standard Deviation; F: inbreeding coefficient.

Table **S2**: **Related to Results. Pairwise F_{ST} values.**

	Iran	Multi-origin ancestry	Poland	Saudi Arabia	Straight Egyptian	Thoroughbred	Turkemen
Bahrain/Syria/Tunisia	0.03	0.03	0.06	0.08	0.12	0.14	0.06
Iran		0.04	0.06	0.08	0.11	0.13	0.05
Multi-origin ancestry			0.02	0.08	0.06	0.13	0.06
Poland				0.11	0.13	0.15	0.08
Saudi Arabia					0.17	0.19	0.11
Straight Egyptian						0.23	0.16
Thoroughbred							0.10

Analysis was conducted using Arlequin v3.5.2.2. The data set included 477 samples and 56,239 SNPs. All pairwise comparisons had a permutation p-value $<$ 1e-4 (20,000 permutations).

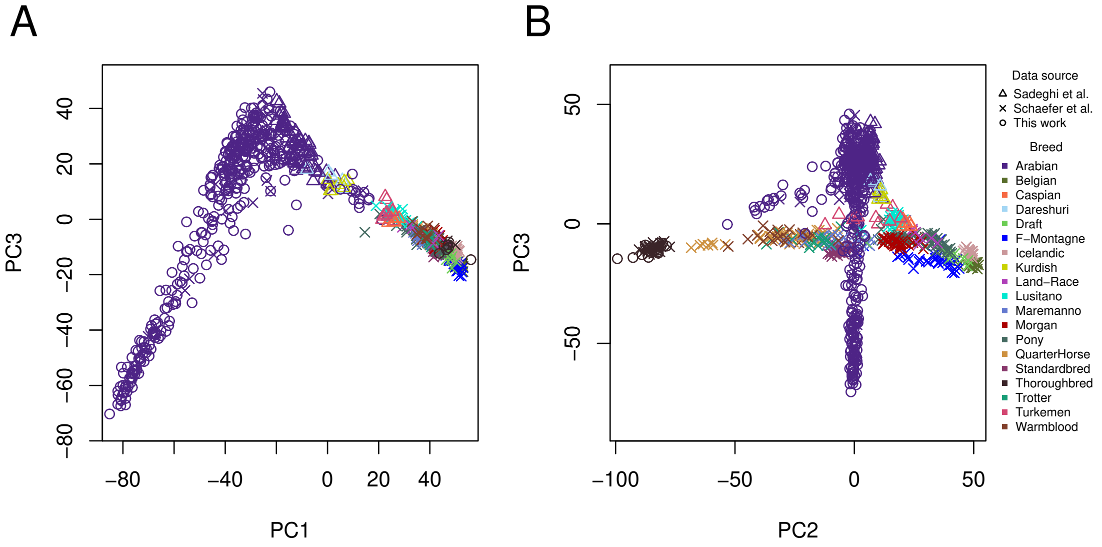

Figure **S2**: **Related to Figure 1A. Principal Component Analysis (PCA) PC3 plots for Figure 1A.** Principal component analysis of 378 Arabian horses sampled in this study among a reference set including samples from 18 additional global breeds from (Sadeghi et al. 2019) and (Schaefer et al. 2017), with symbol shape indicating data source and symbol color indicating breed (data set: 917 samples across 30,967 SNPs). A) PC3 vs. PC1 and B) PC3 vs. PC2. Percent variance explained: PC1 = 5.6%; PC2 = 2.2%; PC3 = 1.8%.

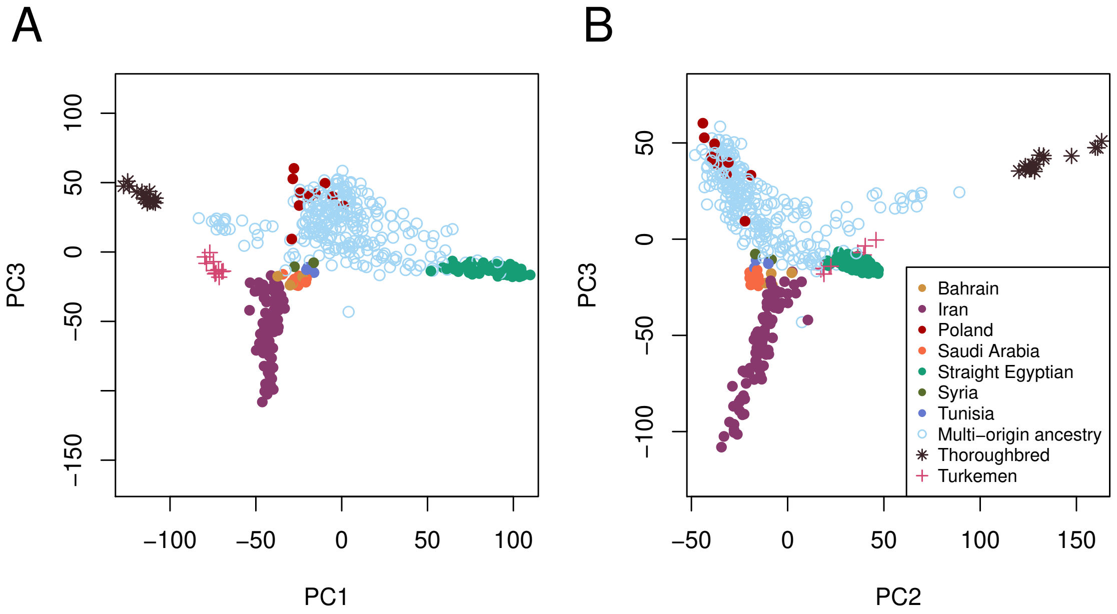

Figure **S3**: **Related to Figure 1B. Principal Component Analysis (PCA) PC3 plots for Figure 1B.** Principal component analysis of 378 Arabian horses sampled in this study with 71 Iran Arabian and 11 Turkemen samples from (Sadeghi et al. 2019), and 17 Thoroughbred samples collected in this study, with symbol shape indicating breed, and symbol color indicating Arabian breed lineage, except for the Thoroughbred and Turkemen groups (data set: 477 samples across 56,239 SNPs). A) PC3 vs. PC1 and B) PC3 vs. PC2. Percent variance explained: PC1 = 4.8%; PC2 = 2.5%; PC3 = 2.1%.

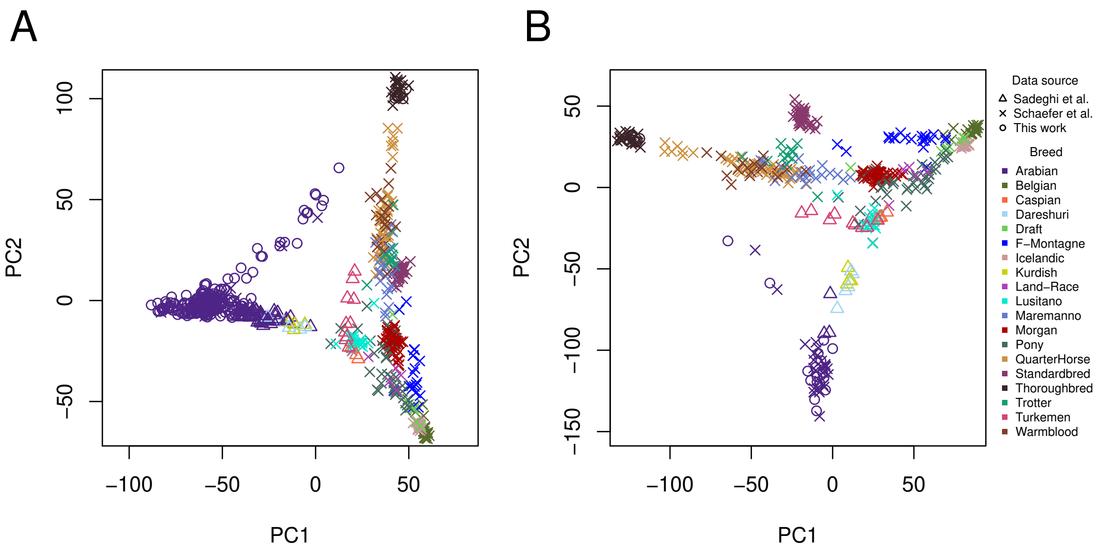

Figure **S4**: **Related to Figure 1A. Principal Component Analysis (PCA) for subsets of the expanded data set.** A) We repeated the PCA in Figure 1A excluding close relatives (pairwise IBD<0.25). This data set included genotype data from 650 individuals across 51,123 SNPs. Qualitatively, the plot presents the same key features as Figure 1A in the main text. Percent variance explained: PC1 = 4.4%; PC2 = 2.3%. B) We repeated the PCA in Figure 1A using a subset of Arabian samples. Figure 1A in the main text includes more Arabian samples than other horse breeds, raising the question whether the inflated spread of the Arabians compared to Thoroughbreds (for example) might in part be driven by this sample size effect. In order to control for this sample size difference, we subsampled the Arabian sample set. We repeated the PCA using several subsets of 20 Arabian samples from this study and Sadeghi et al., 2018, and found that the plots were qualitatively similar. Here we include one such plot, again highlighting the dense cluster of Thoroughbreds, the clear separation of multiple breeds, and the relatively dispersed appearance of Arabians. Percent variance explained: PC1 = 3.3%; PC2 = 2.2%.

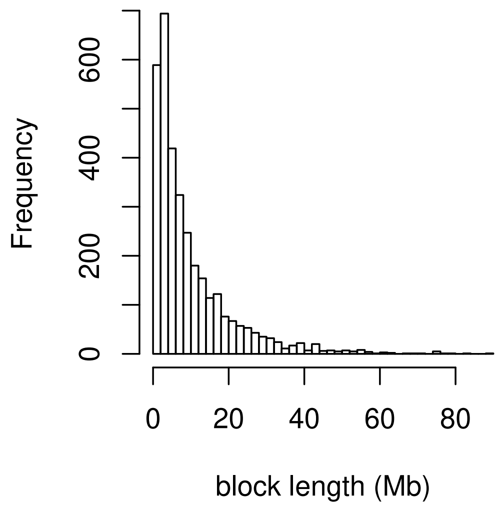

Figure **S5**: **Related to Figure 4. Distribution of RFMix Thoroughbred-assigned local ancestry block lengths.** The observed distribution of Thoroughbred-assigned block lengths suggests multiple introgression events between the Thoroughbred and Racing-type Arabian in both pre-historic and modern history.

Table **S3**: **Related to Methods. Counts of samples collected in this study.**

Breed	Lineage	Chip	WGS
Arabian	Bahrain	4	0
	Iran	9	0
	Multi-origin ancestry	256	3
	Poland	9	2
	Saudi Arabia	12	0
	Straight Egyptian	75	2
	Syria	1	1
	Tunisia	4	0
Standardbred		2	0
Thoroughbred		17	0

Chip: Axiom Equine 670K Genotyping Array; WGS: genotype data from whole genome sequencing

Table **S4**: **Related to Methods. Counts of samples included in expanded data set.**

Breed	Sadeghi et al.	Schaefer et al.		This work	
	Chip	Chip	WGS	Chip	WGS
Arabian	71	21	12	370	8
Belgian	0	21	0	0	0
Caspian	7	0	0	0	0
Dareshuri	5	0	0	0	0
Draft	0	8	0	0	0
F-Montagne	0	0	29	0	0
Icelandic	0	18	0	0	0
Kurdish	7	0	0	0	0
Land-Race	0	6	1	0	0
Lusitano	0	21	0	0	0
Maremanno	0	22	2	0	0
Morgan	0	43	18	0	0
Pony	0	46	0	0	0
QuarterHorse	0	51	0	0	0
Standardbred	0	22	17	2	0
Thoroughbred	0	24	0	17	0
Trotter	0	5	9	0	0
Turkemen	11	0	0	0	0
Warmblood	0	6	17	0	0

Genotype data collected in this work were combined with data from two studies: Schaefer et al., 2017, and Sadeghi et al., 2018. Chip: Axiom Equine 670K Genotyping Array; WGS: genotype data from whole genome sequencing

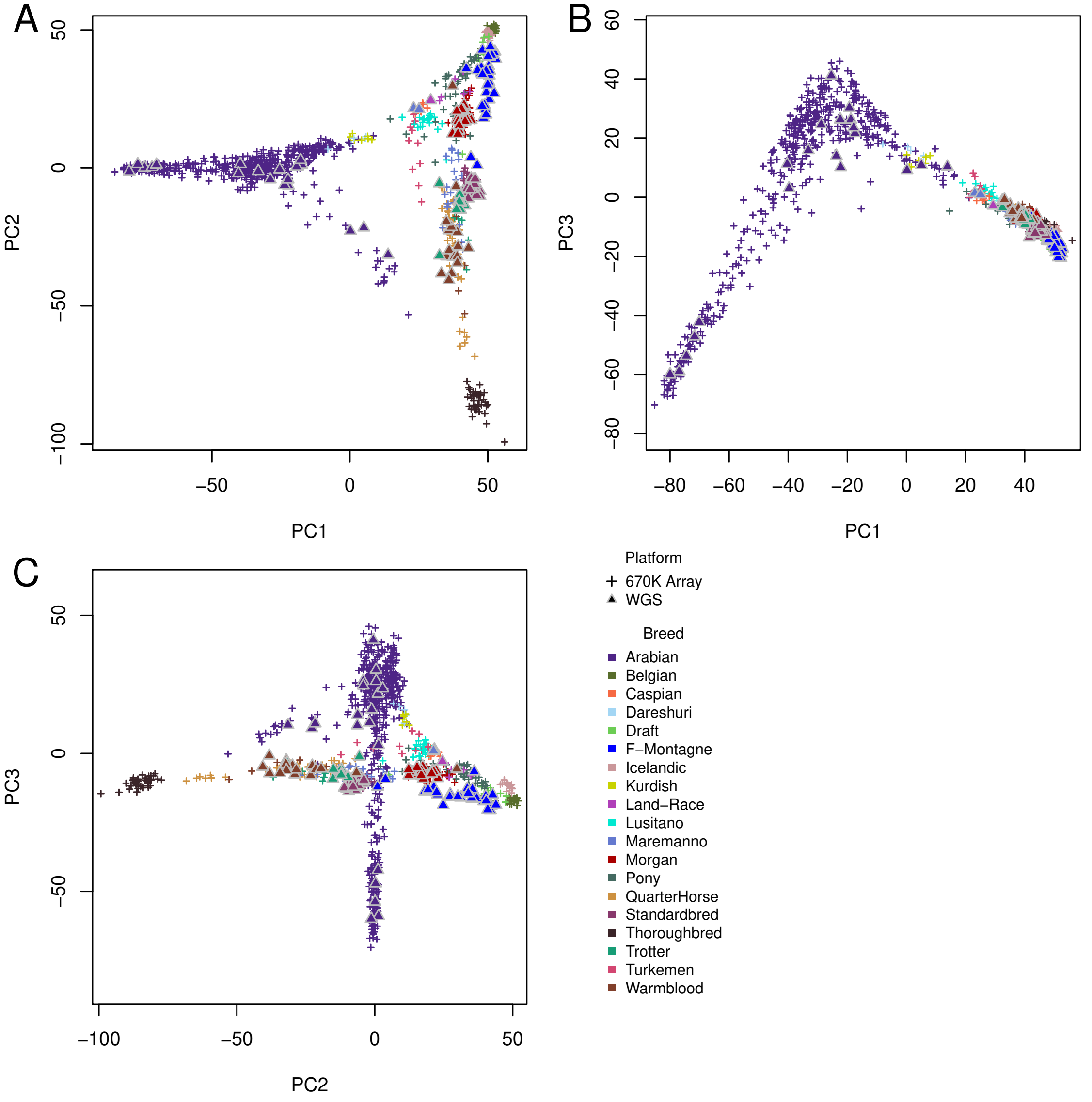

Figure **S6**: **Related to Methods. Principal Component Analysis (PCA) plots for merged data set (Figure 1A) labeled by platform.** Principal component analysis of 378 Arabian horses sampled in this study among a reference set including samples from 18 additional global breeds from (Sadeghi et al. 2019) and (Schaefer et al. 2017), with symbol shape indicating platform and symbol color indicating breed (data set: 917 samples across 30,967 SNPs). A) PC2 vs. PC1, B) PC3 vs. PC1, and C) PC3 vs. PC2. Percent variance explained: PC1 = 5.6%; PC2 = 2.2%; PC3 = 1.8%. Chip: Axiom Equine 670K Genotyping Array; WGS: genotype data from whole genome sequencing

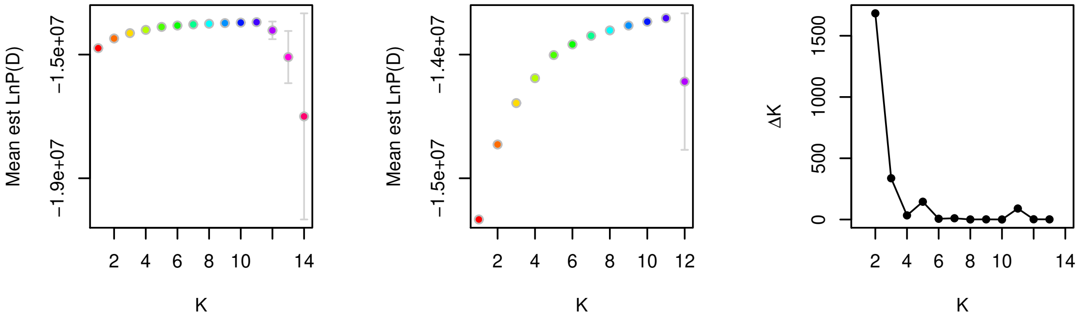

Figure **S7**: **Related to Methods and Figure 3. STRUCTURE results used to select optimal number of clusters K.** The left panel shows mean +/- standard deviation estimated log probability of the data (est LnP(D)) across 5 replicates at each value of K. The center panel is the same plot restricted to K = 1 to 12. The right panel shows the value of Evanno's ΔK statistic at each value K.

Table **S5**: **Related to Methods and Figures 6 and 7. Sample groups used in selection scans.**

Group	Count	Notes
Icelandic	18	Samples from Schaefer et al., 2017
Iran	77	Iran samples from this work and from Sadeghi et al., 2018 with no use defined
Multi-origin	157	Multi-origin ancestry samples with no use defined
Racing (High TB)	17	Racing Arabian samples with higher proportion Thoroughbred-assigned local ancestry
Racing (Low TB)	17	Racing Arabian samples with lower proportion Thoroughbred-assigned local ancestry
Straight Egyptian	34	Straight Egyptian samples with no use defined
Thoroughbred	41	Samples from this work and from Schaefer et al., 2017
Turkemen	11	Samples from Sadeghi et al., 2018
